# Variability in the Occupancy of *Escherichia coli* O157 Integration Sites by Shiga Toxin-Encoding Prophages

**DOI:** 10.3390/toxins13070433

**Published:** 2021-06-22

**Authors:** Scott T. Henderson, Pallavi Singh, David Knupp, David W. Lacher, Galeb S. Abu-Ali, James T. Rudrik, Shannon D. Manning

**Affiliations:** 1 Department of Microbiology and Molecular Genetics, Michigan State University, East Lansing, MI 48824, USA; hende238@gmail.com (S.T.H.); knuppdav@msu.edu (D.K.); 2 Department of Biological Sciences, Northern Illinois University, Dekalb, IL 60115, USA; psingh1@niu.edu; 3 United States Food and Drug Administration, Laurel, MD 20708, USA; David.Lacher@fda.hhs.gov; 4 Janssen Research and Development, LLC, Spring House, PA 19477, USA; abualiga@gmail.com; 5 Michigan Department of Health and Human Services, Lansing, MI 48906, USA; RudrikJ@michigan.gov

**Keywords:** *E. coli*, STEC, Shiga toxin

## Abstract

*Escherichia coli* O157:H7 strains often produce Shiga toxins encoded by genes on lambdoid bacteriophages that insert into multiple loci as prophages. O157 strains were classified into distinct clades that vary in virulence. Herein, we used PCR assays to examine Shiga toxin (Stx) prophage occupancy in *yehV*, *argW*, *wrbA*, and *sbcB* among 346 O157 strains representing nine clades. Overall, *yehV* was occupied in most strains (n = 334, 96.5%), followed by *wrbA* (n = 213, 61.6%), *argW* (n = 103, 29.8%), and *sbcB* (n = 93, 26.9%). Twelve occupancy profiles were identified that varied in frequency and differed across clades. Strains belonging to clade 8 were more likely to have occupied *sbcB* and *argW* sites compared to other clades (*p* < 0.0001), while clade 2 strains were more likely to have occupied *wrbA* sites (*p* < 0.0001). Clade 8 strains also had more than the expected number of occupied sites based on the presence of *stx* variants (*p* < 0.0001). Deletion of a 20 kb non-Stx prophage occupying *yehV* in a clade 8 strain resulted in an ~18-fold decrease in *stx2* expression. These data highlight the complexity of Stx prophage integration and demonstrate that clade 8 strains, which were previously linked to hemolytic uremic syndrome, have unique Stx prophage occupancy profiles that can impact *stx2* expression.

## 1. Introduction

Shiga toxin-producing *Escherichia coli* (STEC), which includes strains belonging to serogroup O157 and other non-O157 serogroups, is a major foodborne pathogen that causes hemorrhagic colitis and hemolytic uremic syndrome (HUS) in some cases [1]. In the United States, STEC were estimated to cause over 265,000 infections each year, leading to more than 3600 hospitalizations and 30 deaths [2]. Although the incidence of O157 infections has decreased over time relative to non-O157 infections, the former were linked to more severe disease and hospitalization [3,4]. Regardless of the serogroup, STEC are defined by their ability to produce one or more antigenically distinct Shiga toxins encoded by genes found on lambdoid bacteriophages [5]. Production of Stx1 and/or Stx2 is most common, though multiple variants have been described [6]. These Stx variants can be found in different combinations that differ in their ability to cause severe disease. Stx2-producing strains, for example, have been correlated with more severe disease outcomes in several epidemiological studies in different locations [7,8,9,10,11]. Moreover, mouse and tissue culture studies have demonstrated that Stx2 is more toxic than Stx1 [12]; however, variation among the Stx2 variants was observed [6]. In addition, the Stx2a lysogenic phage was shown to control a type III secretion system, which is a critical virulence trait found in some STEC O157 strains classified as enterohemorrhagic *E. coli* (EHEC), thereby highlighting its role in the regulation of an important colonization factor as well [13].

The site of prophage integration has also been found to differ across O157 genomes, as Stx-encoding bacteriophages can insert into multiple loci [14,15,16]. Indeed, 59 distinct insertion sites were previously uncovered by examining the sequence of integrase genes in only 13 genomes [17]. The 2006 spinach-associated outbreak strain, TW14359, has a Stx2a prophage inserted within *argW* and an Stx2c prophage in *sbcB* [18]. However, the Sakai and EDL933 genomes have Stx2a prophages in *wrbA* and Stx1a prophages in *yehV* [19,20]. Of particular interest was the finding that *yehV* was occupied with a non-Stx lambda phage in the TW14359 spinach outbreak strain and in other *stx1a*-negative strains [15,18]. Although it is not clear how occupancy by non-Stx prophages impacts virulence in STEC O157, occupancy with Stx-encoding prophages was found to enhance susceptibility to integration by additional phages and impact toxin production [21]. In addition, the site of Stx bacteriophage integration was suggested to be dependent on the host strain, the availability of preferred insertion sites, and the integrase sequence [17,22]. Even though *stx* expression is dependent on prophage induction [23] and variation in *stx* expression levels was observed across O157 strains of different genetic backgrounds [24,25,26], it is not clear whether non-Stx-encoding prophages can differentially affect virulence or disease.

Because Stx prophage integration profiles vary across strains, classifying the Stx-encoding prophage insertion sites across O157 strains has been used for subtyping and epidemiological studies. For instance, one study found that bovine-specific O157 strains and bovine-derived O157 strains, which were more similar to human clinical strains, had distinct Stx prophage insertion site profiles [27]. Greater diversity in the Stx prophage insertion site profiles was also observed between bovine- and human-derived O157 strains. Other methods, such as single nucleotide polymorphism (SNP) genotyping, were used to characterize O157 strains. Indeed, SNP genotyping was used to link Stx prophage insertion site profiles to phylogenetic relationships, examine genotype frequencies, and correlate genotypes with clinical phenotypes [28,29,30]. Our prior study of over 500 O157 strains from humans with varying symptoms uncovered nine O157 clades that varied in their ability to cause clinical illness [28]. More specifically, individuals with HUS were more frequently infected with O157 strains belonging to clade 8 relative to other clades, while clade 7 strains were associated with less severe symptoms. Our follow-up studies demonstrated that O157 strains of clade 8 had an enhanced ability to adhere to bovine mammary epithelial cells and had increased *stx* expression following exposure to epithelial cells compared to clade 2 strains [24,25]. Based on these data, we hypothesized that the distribution and occupancy of certain Stx prophage insertion sites contribute to variation in *stx* expression and is correlated with clinical outcomes. To address this hypothesis, we investigated the occupancy of the four most common Stx prophage insertion sites (*yehV*, *argW*, *wrbA*, and *sbcB*) in 346 O157 strains representing nine clades. We further examined associations between Stx prophage integration profiles and clinical presentation, as well as *stx* expression levels.

## 2. Results

### 2.1. Prophage Occupancy Varied between the Four Insertion Sites and Differed by Clade

Four common insertion sites were evaluated using PCR to determine whether each site was intact or occupied with genetic material. Among the 346 strains examined, most had two (n = 251; 72.5%) or three (n = 74; 21.4%) occupied sites. Only 19 (5.5%) of the strains had one occupied site, while two (0.6%) strains were negative for phage occupancy at all four sites.

**Figure 1:**
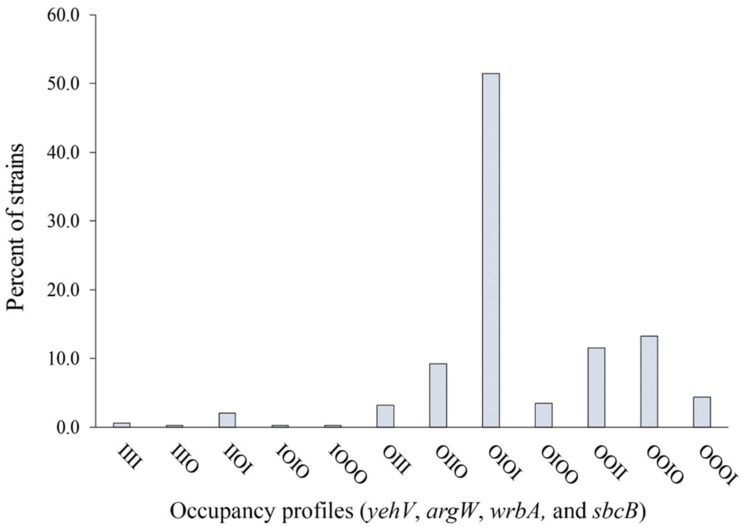
Frequency of Shiga-toxin-encoding bacteriophage integration in *yehV*, *argW*, *wrbA*, and *sbcB*, respectively, among 346 O157 Shiga-toxin-producing *Escherichia coli* strains. O—occupied, I—intact.

The *yehV* site was occupied in most strains (n = 334, 96.5%), followed by *wrbA* (n = 213, 61.6%), *argW* (n = 103, 29.8%), and *sbcB* (n = 93, 26.9%). A binary occupancy profile was utilized to highlight the different insertion site profiles across the strains. Each strain was classified as having intact (I) or occupied (O) sites using the following order of loci: *yehV*, *argW*, *wrbA*, and *sbcB* (Figure 1). In all, 12 occupancy profiles were identified that varied in frequency across the strains (chi-square test for equal proportions = 936.2; *p* < 0.0001). Over half of the strains (n = 178, 51.4%) had a profile of OIOI, indicating occupancy of *yehV*, an intact *argW*, occupancy of *wrbA*, and an intact *sbcB*, respectively.

**Figure 2:**
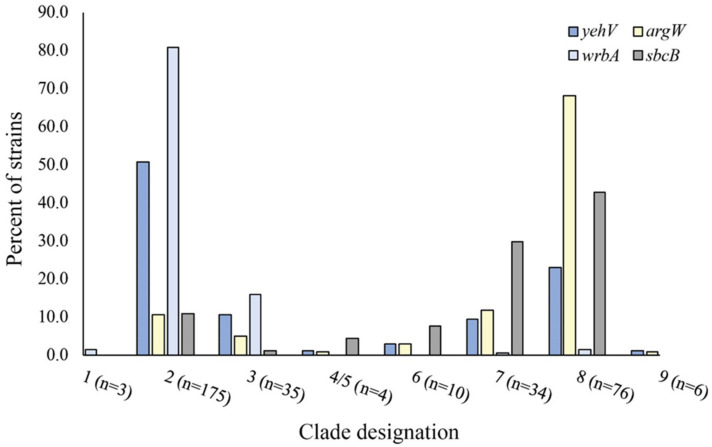
Shiga toxin-encoding prophage occupancy of four insertion sites stratified by clade designation in 343 Shiga toxin-producing *Escherichia coli* O157 strains with clade data available. Note: The clade was not determined for three strains.

Stratification by clade showed differences in the frequency of the occupied sites across lineages. Among the 343 strains previously classified by SNP genotyping [28], most belonged to clade 2 (n = 175), followed by clades 8 (n = 76), 3 (n = 35), 7 (n = 34), 6 (n = 10), 9 (n = 6), and 1 (n = 3). Four strains belonged to clades 4 or 5 and were grouped together based on clustering within the phylogenetic tree. Notably, 80.8% and 50.6% of the clade 2 strains had an occupied *wrbA* site or an occupied *yehV* site, respectively, while clade 8 strains frequently had occupied *argW* (68%) or *sbcB* (42.9%) sites (Figure 2). Those strains belonging to clade 8 were significantly more likely to have occupied *sbcB* and *argW* sites compared to all other clades (*p* < 0.0001) but were less likely to have an occupied *wrbA* site (Fisher’s exact test *p* < 0.0001). However, clade 2 strains, were significantly more likely to have an occupied *wrbA* site relative to strains from the other clades (Fisher’s exact test *p* < 0.0001); only three of these strains had intact sites.

### 2.2. Specific Prophage Occupancy Profiles Predominated in O157 Strains

**Figure 3:**
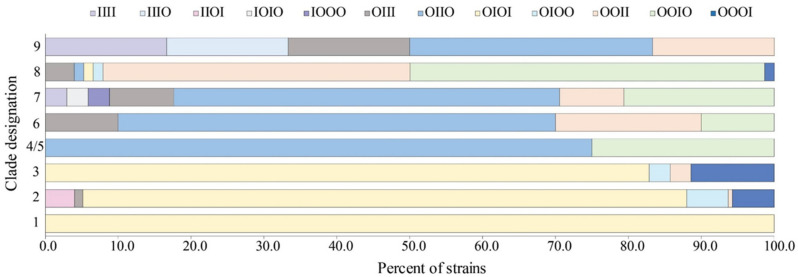
Frequency and occupancy profiles of four Shiga toxin-encoding bacteriophage insertion sites in 343 Shiga toxin-producing *Escherichia coli* strains stratified by clade. Occupancy profiles were assigned a four-letter code based on the occupancy status (intact (I) and occupied (O)) at each site in the following order: *yehV*, *argW*, *wrbA*, and *sbcB*. Frequencies were calculated using the following number of strains within a given clade as the denominator: clade 9 (n = 6), clade 8 (n = 76), clade 7 (n = 34), clade 6 (n = 10), clades 4/5 (n = 4), clade 3 (n = 35), clade 2 (n = 175), and clade 1 (n = 3).

The occupancy profiles, which represented the status of DNA integration at all four insertion sites, were significantly different by clade (Mantel–Haenszel chi-square test = 31.4, df = 1, *p* < 0.0001). Most strains belonging to clade 2, for instance, (n = 145, 82.9%) had the OIOI integration profile with occupied *yehV* and *wrbA* sites and intact *argW* and *sbcB* sites (Figure 3). This OIOI profile also predominated in clades 1 and 3, which comprised strains that were more closely related to clade 2 strains than the other lineages. Twenty additional clade 2 strains had three occupied sites representing profiles OIOO (n = 10, 5.7%) and OOOI (n = 10, 5.7%), while the remaining 10 strains had 3 additional profiles. Occupancy profile OIIO was found most frequently in strains belonging to clades 4, 5, and 6; a high frequency of OOIO was also observed. By contrast, two predominant profiles were identified in the clade 8 strains: OOIO (n = 37, 48.7%) with occupied *yehV*, *argW*, and *sbcB* sites and OOII (n = 32, 42.1%) with occupied *yehV* and *argW* sites. Indeed, most of the 76 clade 8 strains had occupied *yehV* and *argW* loci (n = 70, 92.1%), though four additional profiles (OIII, OIIO, OIOI, and OIOO) were identified. Overall, strains from clades 7 and 8 were the most diverse with seven occupancy profiles each.

### 2.3. Discrepancies Identified between Occupancy Profiles and the Presence of Stx Variants

Comparing the occupancy profile and *stx* status among all 346 strains demonstrated that some strains had either too few or too many occupied sites. More specifically, each strain could harbor up to three Stx-encoding prophages; however, some strains had more or less than the expected number of occupied sites based on the presence of the *stx* variants detected by PCR. The spinach outbreak strain, TW14359, for example, was positive for only *stx2a* and *stx2c*, yet three (*argW*, *yehV*, and *sbcB*) of the four loci were occupied. These data indicate that a non-Stx-encoding prophage was likely located within one of these three Stx prophage integration sites.

Although 60.4% (n = 209) of the 346 strains had the expected number of occupied insertion sites based on the *stx* profile, several discrepancies were observed. Specifically, 10 (2.9%) strains contained fewer than the expected number of occupied sites, whereas 126 (36.4%) strains had more than the expected number of occupied sites. Among these 126 strains, 118 (95.2%) had only one extra site, while six (2.9%) strains had two extra occupied sites. Notably, the clade 8 strains were significantly more likely to have more than the expected number of Stx-encoding prophages compared to strains of other clades (odds ratio (OR): 27.4; 95% confidence interval (CI): 12.89, 58.35). By contrast, clade 2 strains were significantly less likely to contain more than the expected number of occupied sites compared to all other clades (OR: 0.12; 95% CI: 0.07, 0.20).

Because clade 8 strains were previously found to be more common among patients with HUS [28], we also examined occupancy profiles by epidemiological data associated with each case. Among the 296 strains from cases with data available, no association was observed between the possession of an extra occupied insertion site and common symptoms, such as bloody diarrhea or hospitalization. However, it is notable that six of the nine HUS cases were infected with *stx2a*- and *stx2c*-positive clade 8 strains containing more than the expected number of occupied sites with 3 occupancy profiles, OOII (n = 2), OOIO (n = 3), and OIIO (n = 1). Nonetheless, the association between HUS and the presence of an extra occupied site was not significant (Fisher’s exact test *p* = 0.09).

### 2.4. Confirmation of Stx-Encoding Prophages at Specific Occupied Sites

Since 126 strains had an extra occupied insertion site relative to the number of *stx* variants identified, we used long-range PCR to determine which of the four insertion sites were occupied with the Stx-encoding prophages. One strain was missing and could not be evaluated. For this assay, no amplification at a given site indicated that either the integrated prophage contained a different *stx* variant from the one targeted by the PCR assay or that it lacked *stx* altogether. In each case, amplification in the positive control strain ensured that the PCR assays were working. Long-range PCR primers specific for Stx1a-encoding prophages were used only for *yehV*, which was previously found to be important for phages possessing *stx1a* [27,31]. However, the Stx2a/2c primers were used to confirm the occupancy of Stx2a- and Stx2c-encoding prophages in *argW*, *wrbA*, and *sbcB*; the Stx2a- and Stx2c-encoding prophages could not be differentiated in the subset of strains possessing both *stx2a* and *stx2c*. Intact sites were considered negative for Stx-encoding prophages and were not tested using long-range PCR.

Among the 125 strains evaluated, Stx1a-encoding prophages were detected in *yehV* in all but one of the 30 (96.7%) strains containing *stx1a* alone or in combination with *stx2a* or *stx2c* (Table 1). This finding suggests that the Stx1a-encoding bacteriophage preferentially inserted at *yehV* when it was unoccupied. By contrast, Stx2a-encoding prophages were mostly found in *argW* and *wrbA*. Among the 64 strains with *stx2a* alone (n = 38) or in combination with *stx1a*, Stx2a-encoding prophages were detected within *argW* in 34 (53.1%) strains and within *wrbA* in 23 (35.9%). One additional strain had the Stx2a prophage integrated within *sbcB*, while the remaining six strains were *stx2a*-negative at *argW*, *wrbA*, and *sbcB*, indicating that these sites were occupied by non-Stx-encoding prophages. This finding suggests that either *yehV* or another insertion site was harboring the Stx2a prophages in these six strains. The presence of a Stx2a-encoding prophage could not be confirmed for *yehV*, as no positive control strains were available with this occupancy profile. In addition, one strain that was positive for *stx1a* and *stx2a* contained two Stx2a prophages in two different sites (*argW* and *wrbA*); this strain was the only one lacking a Stx1a prophage in *yehV*. No other strains in the collection had more than one copy of the same Stx-encoding prophage in any of the remaining sites evaluated.

Comparatively, the 59 strains with *stx2c* alone (n = 19) or in combination with *stx1a* (n = 2) or *stx2a* (n = 38) mostly had Stx2a/2c prophages integrated within *argW* (55.9%) or *sbcB* (67.8%). Among the 19 strains with only *stx2c*, all but one had Stx2c-encoding prophages in *sbcB*, indicating that these phages preferentially inserted at this site if it was not occupied. However, because the long-range PCR primers could not differentiate between the Stx2a- and Stx2c-encoding bacteriophages, we could not determine which of the two phages were occupying *argW* and *sbcB* among the 38 strains with both *stx2a* and *stx2c*. It is important to note that the sequence region within *stx2B* that differentiated the *stx2a* and *stx2c* variants failed to yield an adequate long-range PCR primer according to the primer software design package (Lasergene PrimerSelect; DNASTAR, Inc.) used. Even though 26 (68.4%) of these strains were occupied by Stx2a/2c prophages in both sites, at least one of the two Stx2a/2c-encoding prophages could not be detected in the other occupied sites in 12 strains. Among all 123 strains with *stx2a* and/or *stx2c* evaluated by long-range PCR, a Stx2a/2c prophage could not be detected within *argW*, *wrbA*, or *sbcB* in 28 (22.8%) strains; most of these strains belonged to clade 8 (n = 16), though some strains represented clades 2 (n = 2), 6 (n = 3), and 7 (n = 5). The clade designation was not known for two strains with missing Stx2c prophages.

### 2.5. Non-Stx-Encoding Prophages Could Affect stx2a Expression

**Figure 4:**
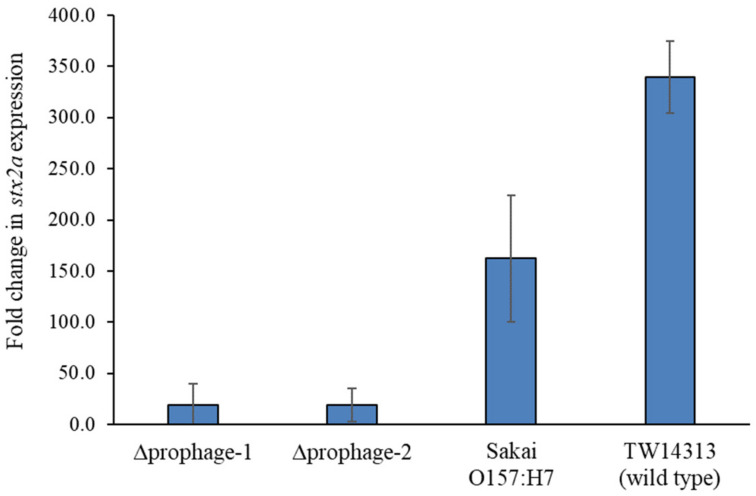
Fold change in *stx2a* expression in two prophage mutants after three hours of growth and induction by mitomycin C. Values were calculated compared to basal growth levels without antibiotics for three hours. RNA was extracted from three separate experiments and qPCR was performed in triplicate. The 16S rRNA (*rrsH*) gene was used for normalization, and fold change values greater than two were considered biologically significant.

To determine whether a non-Stx-encoding prophage inserted within an Stx-encoding bacteriophage integration site affected toxin gene expression, we created two deletion mutants in the O157:H7 strain TW14313. Strain TW14313, which is a *stx1a*-negative, *stx2a*-positive, *stx2c*-negative clade 8 strain, was selected because it was previously shown to have the highest level of *stx2a* gene expression during basal growth [25]. These mutants, *∆yehV_prophage*_TW14313-1_ and *∆yehV_prophage*_TW14313-2_, were both confirmed to have 20 kb deletions of the non-Stx prophages occupying *yehV* in the wild-type strain TW14313 (WT_TW14313_). Following induction with mitomycin C and growth for three hours, the deletion mutants had an average 17.9-fold reduction in *stx2a* expression relative to WT_TW14313_ (Figure 4), indicating that the non-Stx-encoding prophage affected *stx2a* expression in vitro. Relative to the Sakai O157:H7 outbreak strain, an 8.6-fold reduction in *stx2a* expression was also observed for both mutants following induction by mitomycin C after three hours.

## 3. Discussion

*E. coli* O157:H7 strains are highly diverse and have previously been considered ‘phage factories’ [32] when referring to the extensive diversity of prophages that contribute to the evolution of this important foodborne pathogen. The genes encoding the three Stx variants, namely, *stx1a*, *stx2a*, and *stx2c*, are found on distinct lambdoid prophages that preferentially insert into the O157:H7 chromosome at four key loci: *yehV*, *argW*, *wrbA*, and *sbcB* [14,15,16]. In agreement with earlier studies [15,27], *yehV* (96.5%) was most commonly occupied among the 346 O157 strains examined herein, followed by *wrbA* (61.6%) and *argW* (29.8%). Disruption of the *sbcB* integration site was less common (26.9%) and occupancy was mostly restricted to Stx2c-encoding bacteriophages in strains belonging to clades 6, 7, and 8. While the Stx2a-encoding prophages were mostly integrated into *argW* and *wrbA*, as was described in prior studies [15,22], occupancy profiles differed significantly between clades. Clade 8 strains, for instance, mostly contained the Stx2a-encoding prophages at *argW*, whereas the preferred insertion site for Stx2a prophages in clade 2 strains was within *wrbA*. It is therefore likely that the nonrandom occupancy of *argW* and *wrbA* by Stx2a prophages among clades is related to the divergence of phylogenetically distinct O157:H7 subpopulations. While it was shown that bacteriophage insertions within specific sites are dictated by the phage integrases and not the core genome [17], it is possible that more closely related strains will have similar integrases in each site. However, additional studies are needed to confirm this hypothesis.

The identification of a Stx2a prophage within *sbcB* in one clade 2 strain with an intact *argW* locus and the failure to detect several Stx prophages among the four loci demonstrate the complexity of phage integration. Indeed, the disruption of insertion sites by a prophage depends not only on the site availability during lysogeny but also on the complementarity between the phage integrase and the bacterial attachment site [15]. It is particularly notable that we discovered a double lysogen in one clinical strain after performing long-range PCR and amplifying *stx2a* within prophages occupying two different sites, namely, *argW* and *wrbA*. Although a prior study demonstrated that double lysogens can occur following growth in vitro [22], they are not readily detected in clinical strains because most diagnostic tests amplify Stx genes using PCR or detect Stx production via ELISA. While the use of whole-genome sequencing has become more common, it remains difficult to determine the site of Stx prophage occupancy unless longer read sequencing platforms are used [33]. In this study, we detected one Stx2a double lysogen out of the 125 strains evaluated using long-range PCR, indicating that more than one copy was a rare event in our strain population. This strain also lacked *stx2c* but possessed *stx1a*, yet the Stx1a prophage was not confirmed to be occupying *yehV*. An analysis of closed publicly available genomes uncovered a double lysogen in the Japanese O157:H7 strain pv15-279 (GenBank accession number AP018488) that harbors *stx1a*, *stx2c*, and two copies of *stx2a* [34]. In this genome, the *stx1a* and *stx2c* prophages are in *sbcB* and *yehV*, respectively, whereas one *stx2a* prophage is in *yecE* and the other is in *ydfJ*, further highlighting the importance of examining additional insertion sites. Contrary to our findings, the *stx1a* and *stx2c* swapped integration sites in the AP018488 genome, indicating that there may also be variation across locations. However, larger strain collections from distinct geographic regions need to be evaluated for confirmation.

Among the 123 strains evaluated for the presence of Stx2a or Stx2c prophages using long-range PCR, we failed to detect the prophages in *argW*, *wrbA*, or *sbcB* in 28 (22.8%) strains. For this subset of strains, we cannot rule out the possibility that the Stx2a/2c prophages were integrated within *yehV*, at least for the 26 *stx1a*-negative strains. Confirmation of Stx2a prophages occupying *yehV* could not be evaluated since a control strain was not available, thereby limiting our ability to develop a PCR assay that could target these prophages within this site. Regardless, a prior study identified truncated phages occupying *yehV* in most of the 35 *stx1a*-negative O157:H7 strains examined [15]; hence, we expect that the Stx2a/2c prophages were not occupying this locus and they, too, may possess truncations that can only be confirmed by sequencing. It is therefore more likely that the Stx2a/2c prophages were integrated within the *yecE* or *Z2577* insertion sites, which were not examined but were previously shown to be occupied by Stx prophages at low frequencies [22]. The same is true for the 59 additional insertion sites previously detected using whole-genome sequencing [17]. Clearly, a more comprehensive genomic analysis of this strain subset is needed to better define the Stx prophage distribution. Such analyses would require long-read sequencing methods to define the Stx prophage integration sites and differentiate the Stx2a/Stx2c prophages; *stx2a* and *stx2c* differ by 11 SNPs (three amino acids) and could not be distinguished using our long-range PCR assays. 

O157 strains belonging to clade 8 were linked to HUS more frequently than other lineages [28]. Although the underlying mechanism for this association is not clear, our prior work implies that increased adherence potential and expression of *stx2a* in clade 8 strains may confer pathogen persistence in the gut and toxin accumulation that precipitates progression to severe disease [24]. It is therefore noteworthy that the strains containing more than the expected number of occupied insertion sites were significantly more likely to belong to clade 8 than any other clade. Indeed, the presence of non-Stx prophages was documented. While they were suggested to represent either complete, inducible phages or non-inducible, remnant, cryptic or residual phages [35], their role in pathogenesis is not clear and likely differs across strains. In the O157 Sakai genome, for instance, 18 distinct prophages were identified; most contained numerous genetic defects, though some were inducible [36]. The increased number of occupied Stx-encoding bacteriophage insertion sites relative to the number of Stx prophages further highlights the plasticity of the *E. coli* O157 genome and a divergent evolutionary trend of this pathogen. Prior studies documented recombination between prophages within the same bacterial genome [32,37], which was suggested to occur between homologous regions among co-existing prophages [35]. However, differentiating between these Stx and non-Stx prophages using whole-genome sequencing is difficult given that contig assembly programs cannot always distinguish them from each other [35]. Through the use of multiple PCR-based assays described herein, we were able to determine which insertion sites harbored the Stx genes in most of the strains examined. Such tools are particularly useful for epidemiological studies and the characterization of large strain collections.

Although we did not observe a significant association between the presence of extra occupied integration sites and HUS or other symptoms, deletion of the non-Stx prophage occupying *yehV* in a strain with high levels of toxin expression resulted in reduced *stx2a* expression following antibiotic induction (Figure 4). Although *stx2a* induction was examined following mitomycin C exposure, other clinically relevant antibiotics could be assessed in the future. Genomic analysis of strain TW14313 (GenBank accession number AOMX00000000.1) indicated that the non-Stx phage integrated within *yehV* possessed multiple genes that are important for *stx* expression. Examples include genes encoding the antitermination protein Q (gene #3035), which regulates the expression of early and late phage genes, and the bacteriophage regulatory protein, CII (gene #3045), which activates the CI repressor to prevent the transcription of genes required for the lytic cycle. The gene encoding the CI repressor was also found (gene #3047). All of these genes were previously implicated in Stx production in *E. coli* O157 [38], as has the genotype of a given Stx prophage [39]. Collectively, these data strongly suggest that *stx* expression in some strains is regulated in part by former Stx-encoding prophages that have lost *stx* genes but have retained regulatory genes that are important for *stx* transcription. This finding is in line with two prior studies demonstrating that genes on different prophages can act in *trans* to affect toxin production [21,40]. Further support for prophage interactions within a genome comes from the observation that the type III secretion system was controlled by genes found on the Stx prophage in conjunction with those found on non-Stx prophages encoding type III effector proteins [13]. How these non-Stx prophage regulatory genes affect Stx production following the induction of Stx-encoding prophages integrated into different loci around the chromosome is not clear and requires further study. Moreover, enhancing our understanding of the genetic composition of non-Stx prophages in clade 8 strains is needed to determine the extent that these prophages contribute to variations in virulence.

## 4. Materials and Methods

### 4.1. Bacterial Strains

A total of 346 O157 strains, previously evaluated using SNP genotyping that targeted 96 SNP loci and *stx* profiling [28], were selected for characterization. Most strains (n = 326) were isolated from clinical cases in Michigan between 2001 and 2006 [41], while 23 strains were reference strains from different sources and locations available through the STEC Center at Michigan State University (www.shigatox.net, assessed on 21 January 2021). Following overnight growth in Luria–Bertani (LB) broth at 37 °C, DNA was extracted using the Puregene DNA isolation kit (Qiagen, Valencia, CA, USA).

### 4.2. PCR Assays to Detect Prophage Integration

Four of the most common Stx-encoding bacteriophage insertion sites, namely, *yehV*, *argW*, *wrbA*, and *sbcB*, were examined for prophage occupancy in the 346 strains using three PCR assays. Primers for each assay were designed using the following *E. coli* O157:H7 reference genomes: Sakai (RIMD 0509952) [19], EDL933 [20], and TW14359 from the 2006 North American spinach outbreak [18,28] (Table A1). With the conserved housekeeping gene *mdh* as an internal positive control, the PCR conditions were as follows: 10 min at 94 °C; 20 cycles of 1 min at 92 °C, 1 min at 57 °C, and 1 min at 72 °C; followed by 5 min at 72 °C. 

**Figure 5:**
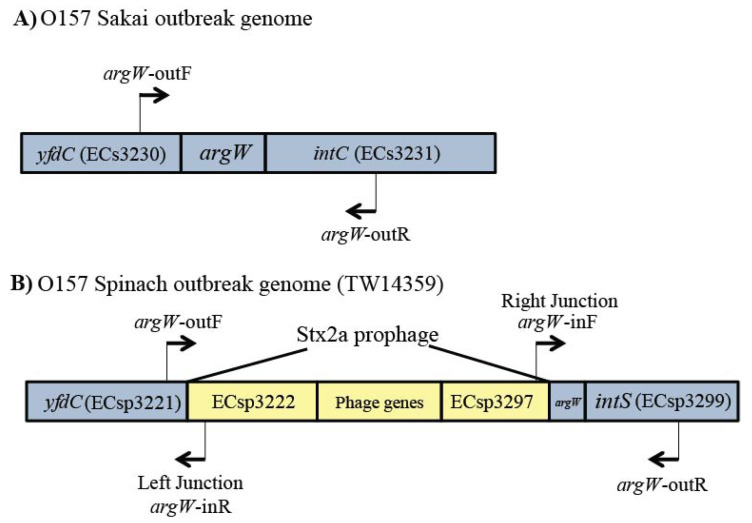
Representation of the *argW* Shiga toxin-encoding bacteriophage integration site in two O157:H7 outbreak strains. (**A**) Primers in adjacent genes allowed for the amplification of an intact *argW* integration site, while (**B**) the combination of four primers allowed for the amplification of the right and left junctions of *argW* when it was occupied by a Stx2a prophage. Drawing is not to scale.

PCR was first performed by utilizing primers targeting the genes flanking each integration locus to amplify the full gene and determine whether it was intact in each strain. In silico analysis of the three genomes demonstrated that the use of these flanking primers yielded varying-sized products. In the Sakai genome, for example, *argW* (ECs5488) was intact and not interrupted by a prophage, resulting in the amplification of a 527 bp PCR product spanning the region from *yfcD* (ECs3230) to *int*C (ECs3231) (Figure 5A). However, in silico genome analysis of TW14359 identified a Stx2a prophage integrated within *argW*, and therefore, no amplification of the intact *argW* gene was observed using the *argW*-outF and *argW*-outR primers. All primers were designed using the Lasergene PrimerSelect software package (DNASTAR, Inc., Madison, WI, USA).

To confirm the prophage/DNA occupancy within genes that were not classified as intact using the initial PCR assay, multiplex PCR was used to amplify the junctions between the prophage and flanking genes (Figure 5B). The same PCR conditions were used (10 min at 94 °C; 20 cycles of 1 min at 92 °C, 1 min at 57 °C, and 1 min at 72 °C; followed by 5 min at 72 °C) with *mdh* as an internal positive control. Prophage occupancy within *argW* in strain TW14359, for instance, resulted in the amplification of the left junction (*argW*-outF to *argW*-inR, 442 bp) and the right junction (*argW*-inF to *argW*-outR, 1395 bp). Primers for the left junction utilized the same forward primer as the intact PCR assay and another primer that targeted the region between *yfcD* and ECsp3222, a conserved hypothetical protein located within the Stx2a prophage (Table A1). The reverse primer within *intS* (ECsp3299) was also used for the right junction multiplex PCR assay combined with a primer targeting the prophage integrase, namely, *intW* (ECsp3297), located within the Stx2a prophage. Amplification of both junction site fragments indicated that a prophage was present within a given integration site.

### 4.3. Frequency of Prophage Occupancy Profiles

Based on the results of the prophage integration PCR assays, each strain was assigned an occupancy profile. These binary profiles were based on the presence of intact (I) or occupied (O) insertion sites in the following order: *yehV*, *argW*, *wrbA*, and *sbcB*. The distribution of occupancy profiles was examined via O157 clade designation, as determined using SNP genotyping and *stx* profiles. Differences in the frequencies of clinical symptoms and disease presentation were examined among occupancy profiles for the 326 clinical isolates recovered in Michigan using the likelihood chi-square test or Fisher’s exact test for sample sizes less than five. Associations were described using the odds ratio (OR) with 95% confidence intervals (95% CI).

### 4.4. Long-Range PCR to Confirm Insertion Site Occupancy with Stx Prophages

Long-range PCR assays were developed using the long and accurate (LA) *Taq* polymerase (Takara Bio, Inc., Ann Arbor, USA) to confirm that specific Stx prophages were occupying integration sites, as determined using the junction multiplex PCR assays. One primer was designed to target the genes flanking each insertion site and the second primer targeted either *stx1a* or *stx2a/2c* present within the Stx prophages. The following PCR conditions were used: 30 s at 94 °C; followed by 30 cycles of 30 s at 94 °C, 30 s at 65 °C, and 20 min at 68 °C; with a final soak at 72 °C for 10 min. Amplicon sizes varied depending on the type of Stx-prophage and insertion site, ranging between 19.4 kb for *yehV* and 26.3 kb for *sbcB*. A previously described PCR-based RFLP assay [28] was used to differentiate between *stx2a* and *stx2c*; however, the precise locations of the Stx2a- and Stx2c-prophages could not be determined among the 52 strains containing both types due to the high level of similarity between the *stx2a* and *stx2c* variants. The Sakai and TW14359 O157 strains were used as controls. A lack of amplification using this assay indicated that either the integrated prophage contained a genetically distinct *stx* or lacked *stx* altogether.

### 4.5. Deletion of a 20 kb Non-Stx Prophage in TW14313

A 3-step PCR amplification protocol was used to amplify the upstream and downstream junctions of *yehV*, which is a site within TW14313 occupied by a non-Stx prophage, and a kanamycin resistance cassette. Primers were designed to amplify two segments adjacent to the non-Stx prophage, which was targeted for excision. The following primers were used: forward_upstream—AAGTGGCGTTGCTTTGTGATA, reverse_upstream—CTGGACGATCTTCGTCGATTC CAATTCACTGTTCCTTGCA, forward_downstream—AGTACATCCGCAACTGTCC ATTGCTGAAACCGCAACGGACA, and reverse_downstream—GTTGTCGATCCAGC GTTTG. The three fragments were pooled and amplified to yield a product containing the upstream and downstream regions flanked by the kanamycin cassette. Phage-λ-Red-mediated recombinase was used to promote homologous recombination, as described previously [42], with the help of pSIM5, which is a temperature-sensitive plasmid expressing key λ Red proteins [43]. Two deletion mutants (∆*prophage*_TW14313-1_; ∆*prophage*_TW14313-2_) were confirmed to be negative for the 20 kb region using PCR and Sanger sequencing.

### 4.6. Quantifying stx2a Expression

The expression of *stx2a* was examined in the two ∆*prophage*_TW14313_ mutants for comparison to the TW14313 wild-type strain and the Sakai O157:H7 outbreak strain. Gene expression was quantified following growth LB broth and after induction with 25 ng/mL mitomycin C (Sigma-Aldrich, Inc. St. Louis, USA) using a modified version of a published protocol [44]. Briefly, overnight cultures grown in LB at 37 °C with shaking were diluted in LB to an OD_600_ of 0.2–0.3. At time T_0_, 2 mL of culture was collected for RNA isolation and the remaining culture was divided into two tubes; mitomycin C (25 ng/mL) was added to one tube for comparison. After incubation at 37 °C for three hours, a 2 mL culture was collected for RNA isolation. RNA was extracted using the RNeasy kit (Qiagen, Valencia, CA, USA). Expression levels were compared between each mutant to the WT_TW14313_ strain following induction by mitomycin C relative to growth in LB alone after three hours. RNA was isolated in three separate experiments and qPCR was performed in triplicate using published *stx2B* primers, while the 16S rRNA (*rrsH*) gene was used for normalization to quantify mRNA transcription levels, as described in [24,45]. Relative gene expression was determined based on the use of an SYBR green qPCR assay with the following conditions: 95 °C for 3 min, followed by 39 cycles of 95 °C for 10 s and 55 °C for 30 s. Fold change was calculated using the ddCt method [46]; values greater than two were considered biologically significant.

## Figures and Tables

**Table 1:** Distribution of Stx-encoding bacteriophages by insertion site, as determined using long-range PCR among 125 Shiga toxin-producing *Escherichia coli* O157 strains.

		*yehV*		*argW*		*wrbA*		*sbcB*	
*stx* Profile	Profile ^1^	Stx1a Phage	Non-Stx1a Phage	Stx2a/2c Phage	Non-Stx2a/2c Phage	Stx2a/2c Phage	Non-Stx2a/2c Phage	Stx2a/2c Phage	Non-Stx2a/2c Phage
*1a* (n = 2)	OIOI	2	0	-	-	0	2	-	-
*1a*, *2a* (n = 11)	OIOO	8	0	0	0	8	0	0	8
	OIOO	1	0	-	-	0	1	1	0
	OIOO *	2	0	-	-	0	2	0	2
*1a*, *2a* (n = 15)	OOOI **	0	1	1	0	1	0	-	-
	OOOI	11	0	0	11	11	0	-	-
	OOOI	3	0	3	0	0	3	-	-
*1a*, *2c* (n = 2)	OOIO	1	0	1	0	-	-	0	1
	OOIO	1	0	0	1	-	-	1	0
*2a* (n = 3)	OIOI	0	3	-	-	3	0	-	-
*2a* (n = 31)	OOII *	0	5	0	5	-	-	-	-
	OOII	0	26	26	0	-	-	-	-
*2a* (n = 4)	OOIO	0	4	4	0	-	-	0	4
*2a*, *2c* (n = 1)	OIOO *	0	1	-	-	0	1	0	1
*2a*, *2c* (n = 37)	OOIO *	0	2	0	2	-	-	0	2
	OOIO *	0	6	6	0	-	-	0	6
	OOIO	0	26	26	0	-	-	26	0
	OOIO *	0	3	0	3	-	-	3	0
*2c* (n = 1)	IOOO	-	-	0	1	0	1	1	0
*2c* (n = 17)	OIIO *	0	9	-	-	-	-	0	9
	OIIO	0	8	-	-	-	-	8	0
*2c* (n = 1)	OOIO	0	1	0	1	-	-	1	0
Total	(n = 125)	29	95	67	24	23	10	41	33

^1^ Occupancy profile refers to the pattern of occupied (O) and intact (I) insertion sites for *yehV*, *argW*, *wrbA*, and *sbcB*. * Stx2a/2c-bacteriophages were not found in any of the sites evaluated. ** Two Stx2a-encoding bacteriophages were found in *wrbA* and *argW*; the Stx1a phage was not found within *yehV*.

## Data Availability

All data supporting the results can be found within the manuscript. Genome accession numbers are provided for publicly available genomes.

## Appendix A

**Table A1:** The PCR primers used to amplify genes that are commonly interrupted by Shiga toxin (Stx) prophages to determine whether they were intact or occupied in 346 O157 strains. Primers were designed to amplify complete/intact genes, as well as the left and right junctions of each gene that was occupied with Stx prophages or prophage remnants. Long-range PCR primers were used to amplify the region between the Stx genes and the sequence flanking the associated Stx prophages.

Gene	Product Target	Primer Name	Primer for Sakai	Primer for TW14359 ^1^	Sequence 5′ to 3′	Size
*yehV*	Intact *yehV*	*yehV*-outF	ECs2937	ECSP2931	GAAGCAGCAGCAACACCAGATTA	543 bp
		*yehV*-outR	ECs3014	ECSP2998	ATTGCCGCTTTGCAGGTAGGT	
	*yehV* left junction	*yehV*-outF				607 bp
		*yehV*-inR	ECs2939	ECSP2932	ACAGCACCGTTTGGCATTTTAG	
	*yehV* right junction	*yehV*-inF	ECs3013	ECSP2997	GAAGCAGAGGGAATATGGGAGAACT	654 bp
		*yehV*-outR				
	*yehV* with Stx1a phage	*yehV*long-F	ECs2974 (*stx1a*)	Absent	CAAACAAATTATCCCCTGTGCCACTA	19.4 kb
		*yehV*long-R	ECs3015 (*yeh*W)	ECSP3000 (*yeh*W)	AGTTGCGAAAGGTATGGGAATGAGTC	
*argW*	Intact *argW*	*argW*-outF	ECs3230	ECSP3221	CTGGCCCTTCGCACTACCTACTT	527 bp
		*argW*-outR	ECs3231	ECSP3299	TCTTTCGGCTTTGCTGCTTCA	
	*argW* left junction	*argW*-outF				442 bp
		*argW*-inR	Absent	ECSP3222	GGATCGGTTTAACCGCGAGTAC	
	*argW* right junction	*argW*-inF	absent	ECSP3297	TTAGCCGACGAACGATCAATAGGT	1395 bp
		*argW*-outR				
	*argW* with Stx2a phage	*argW*long-F	Absent	ECSP3253 (*stx*2*a*)	GAGGGGTCGATATCTCTGTTCGTATACTATTTA	25.6 kb
		*argW*long-R	ECs3231 (*int*C)	ECSP3299 (*int*S)	ATTTCAAGGCCGCCGATGATAGGT	
*wrbA*	Intact *wrbA*	*wrbA*-outF	ECs1159.5 ^2^	ECSP1174	TACCGCCGCGAACCTGTG	1118 bp
		*wrbA*-outR	ECs1252	ECSP1175	CTTTGGCGCGATTTTACTCAATG	
	*wrbA* left junction	*wrbA*-outF				605 bp
		*wrbA*-inR	ECs1160	Absent	CCAGCGCCAGCATGGTCTAC	
	*wrbA* right junction	*wrbA*-inF1	ECs1251	Absent	TTTTTCCCTCGCCCATAACCTAT	1445 bp
		*wrbA*-outR				
	*wrbA* with Stx2a phage	*wrbA*long-F	ECs1158 (*agp*)	ECSP1172 (*agp)*	GAAAGTCGGCAACTCGCTGGTAGA	22.4 kb
		*wrbA*long-R	ECs1205 (*stx*2A)	Absent	AAGTCTATCGTAAACTCCCGGGAATA	
*sbcB*	Intact *sbcB*	*sbcB*-outF	ECs2812	ECSP2683	TGACCGGTGGTGTAATCCATCA	636 bp
		*sbcB*-outR	ECs2813	ECSP2762	CGGTACGGTAAAAAGCGAGTGAAT	
	*sbcB* left junction	*sbcB*-outF				1441 bp
		*sbcB*-inR	Absent	ECSP2685	GTTTGTGATTTCGCGCTGTAGGT	
	*sbcB* right junction	*sbcB*-inF	Absent	ECSP2761	CGCTTTGCGCTGAGCCTCTAT	1588 bp
		*sbcB*-outR				
	*sbcB* with Stx2c phage	*sbcB*long-F	Absent	ECSP2722 (*stx*2cB)	CGGCGCCATTGCATTAACAGAAACTA	26.3 kb
		*sbcB*long-R	ECs2815 (*yeeE*)	ECSP2764 (*yeeE*)	TTGCGTTAATTCAGGCGGGCCTACT	

Note: Blank spaces indicate that the same primer was used for amplification of the junctions as was noted for each intact gene. ^1^ *yehW*, *intS*, *agp*, and *yeeE* from the TW14359 genome have 100% nucleotide similarity to the corresponding genes in the Sakai genome. ^2^ The ECs1159.5 primer targeted the sequence between ECs1159 and ECs1160 corresponding to the *N*-terminal portion of the interrupted *wrbA* gene.
